# Comparing Ovarian Radiation Doses in Flat-Panel and Conventional Angiography During Uterine Artery Embolization: A Randomized Clinical Trial

**DOI:** 10.5812/iranjradiol.13264

**Published:** 2013-08-30

**Authors:** Kavous Firouznia, Hossein Ghanaati, Aliakbar Sharafi, Firouze Abahashemi, Hassan Hashemi, Amir Hossein Jalali, Madjid Shakiba

**Affiliations:** 1 Advanced Diagnostic and Interventional Radiology Research Center (ADIR), Tehran University of Medical Sciences, Tehran, Iran; 2 Medical Physics Department, Iran University of Medical Sciences, Tehran, Iran

**Keywords:** Uterine Artery, Embolization, Therapeutic, Radiation, Angiography

## Abstract

**Background:**

Uterine artery embolization (UAE) is a minimally invasive procedure performed under fluoroscopy for the treatment of uterine fibroids and accompanied by radiation exposure.

**Objectives:**

To compare ovarian radiation doses during uterine artery embolization (UAE) in patients using conventional digital subtraction angiography (DSA) with those using digital flat-panel technology.

**Patients and Methods:**

Thirty women who were candidates for UAE were randomly enrolled for one of the two angiographic systems. Ovarian doses were calculated according to in-vitro phantom study results using entrance and exit doses and were compared between the two groups.

**Results:**

The mean right entrance dose was 1586±1221 mGy in the conventional and 522.3±400.1 mGy in the flat panel group (P=0.005). These figures were 1470±1170 mGy and 456±396 mGy, respectively for the left side (P=0.006). The mean right exit dose was 18.8±12.3 for the conventional and 9.4±6.4 mGy for the flat panel group (P=0.013). These figures were 16.7±11.3 and 10.2±7.2 mGy, respectively for the left side (P=0.06). The mean right ovarian dose was 139.9±92 in the conventional and 23.6±16.2 mGy in the flat panel group (P<0.0001). These figures were 101.7±77.6 and 24.6±16.9 mGy, respectively for the left side (P=0.002).

**Conclusion:**

Flat panel system can significantly reduce the ovarian radiation dose during UAE compared with conventional DSA.

## 1. Background

Uterine fibroids are the most common tumors of the female pelvis, and uterine artery embolization (UAE) has been accepted as an alternative modality for the treatment of symptomatic uterine fibroids (1-4). UAE is considered as a minimally invasive procedure performed under fluoroscopy. In this method, the interventional radiologist embolizes the uterine or fibroid artery by embolization materials such as polyvinyl alcohol (PVA) particles via fluoroscopically-guided angiographic techniques. All fluoroscopically-guided interventional techniques are accompanied by radiation exposure. Considering the location of radiation (pelvic region containing ovaries) and the patients’ age (fertility age), measuring the radiation dose to the ovaries is highly important in UAE.

The advent of flat panel detectors results in the increase of image quality as well as significant reduction in the radiation dose (5-7). The benefit of flat-panel angiography that delivers high-resolution images has been evaluated in previous studies (8).

The efficacy of this system in reducing the dose in interventional procedures has been reported in many studies (8-10). To our knowledge, there is no other study comparing the ovarian’s radiation dose in the flat panel and conventional angiography unit in the UAE procedure.

## 2. Objectives

In this study, we will evaluate and compare the radiation dose to ovaries during UAE in flat-panel and conventional angiography units.

## 3. Patients and Methods

Because it is not possible to put thermoluminescent dosimeter (TLD) chips inside the body to directly measure the ovarian dose, the study was performed in two steps (in-vitro and in-vivo). The results of the in-vitro study were used to calculate ovarian dose in the in-vivo study. The TLDs used for this study were LiF: TLD-100, manufactured by Bircon/Harshow (city/country), packed in groups of three. Reading of the TLD-100 chips was carried out by the TLD reader system model Harshaw-3500.

### 3.1. In-Vitro Study

In this step, the radiation attenuation curves were derived in an anthropomorphic phantom for both conventional DSA (Advantx, GE Medical Systems, Illinois, USA) and flat panel (Innova 4100, GE Medical Systems, Illinois, USA) angiography systems used in this study for calculating the depth dose according to entrance surface dose (ESD). Ten batches containing TLD-100 chips were put on the posterior surface (entrance of X-rays) and in different depths of the phantom at the right and left ovarian regions and mid-point of the phantom. Each batch containing three chips of TLD-10 was used in each location to improve the accuracy and the mean of three TLD chip readings was used to measure the radiation dose of that location. The configuration of angiography systems such as tube-table distance and patient to image receptor distance was set for routine UAE procedures.

As a fixed combination of these two parameters, we set the focal spot-detector distance for both systems at 95 cm. Medium size field of view (FOV) was used in this study for in-vitro and in-vivo steps that was fixed as 30 cm in the conventional DSA and 32 cm in the flat panel system.

The high beam filter used consisted of 1 mm aluminum and 0.1 mm copper for conventional DSA system. For the flat panel system, the high beam filter used was 0.2-0.3 mm aluminum and was selected automatically by the system. The exposure parameters were similar to the routine UAE procedure (pelvic adult program in the DSA system and abdomen pelvic adult program in the flat panel system). The phantom and TLDs were exposed by 20 minutes fluoroscopy and 40 spot images. Then the TLD chips were removed and read by the TLD-reader system. This step was repeated for each location of the right and left ovaries and for the midpoint of the phantom and for both angiography systems separately to achieve the radiation attenuation function for each angiography system. The dose-depth relation was calculated by Excel program using data of ESD and different depth doses. We used the exponential curve fitting and formulation; the depth was considered as the independent variable and the measured dose was considered as the dependent variable. For all fittings, R^2 ^of model was calculated. The equations are as follows: For the DSA system;

Dx=D20 e-0.19x + 4.07 with R^2^= 0.978 and for the flat panel system Dx=D20 e-0.09x+1.87 with R^2 ^= 0.971.

X=the depth of organ or tissue of interest from the posterior surface.

Dx = Absorbed Dose (mrad) at depth x

D20 = Absorbed Dose (mrad) at AP surface of patients or the phantom

### 3.2. In-Vivo Study

Thirty patients (fifteen patients for each system) who were referred for UAE to the medical imaging center affiliated to our university hospital and were approved as patients requiring UAE according to guidelines for the procedure (11) were enrolled in this parallel designed clinical trial and were randomly assigned to each of the angiography systems. For sample size, considering type I and II statistical errors equal to 5% and 10%, respectively and conducting a pilot study, we calculated the sample size of 11 patients for each group and finally we considered 15 patients for each group. Using block randomization method the patients were assigned to the groups. Only the study coordinator was aware of the allocation sequence and assigned each patient to her group after enrollment to the study by the care provider physician. The physicist who performed dosimetry and the statistical analyzer was blinded to the patient’s group.

The study was approved by the Institutional Review Board and informed consent was obtained from each patient before the study. The additional costs of this project were funded by the research department of Tehran University of Medical Sciences.

Patient information, including age, height, weight, anterio-posterior thickness of the pelvis in the supine position, depth of the right and left ovaries from the posterior surface of the body (measured by MRI axial images) were filled in a data collection form before the study.

### 3.3. Angiography

Before the study, we performed the quality control of our devices with KVP meter and milliampere meter. KVP were defined manually according to the antero-posterior (AP) thickness of the pelvis of each patient and the image view (AP or oblique view) that was in the range of 75-95 KV. The milliampere (mA) tube filament for fluoroscopy and milliampere-second (mAs) for each exposure of spot images were automatically set by the angiography system.

All patients underwent catheterization through a right femoral approach. Aortography was performed with a pigtail catheter before pelvic arteriography. The catheter was placed in the abdominal aorta at the level of the renal arteries and selective catheterization of the uterine arteries was performed.

Then a 4-French cobra-shaped catheter was positioned beyond the junction of the descending and horizontal portions of each uterine artery. Embolization was performed by injection of PVA particles under fluoroscopic control. To avoid retrograde reflux of the particles and infiltration to other internal iliac artery side branches, the injection was stopped when the arterial flow ceased. When an anastomosis was encountered between the uterine and ovarian arteries, the catheter tip was placed in a position distal to the anastomosis. Postembolization angiography was performed for evaluation of redistribution.

### 3.4. In-Vivo Dosimetry

**Figure 1.**
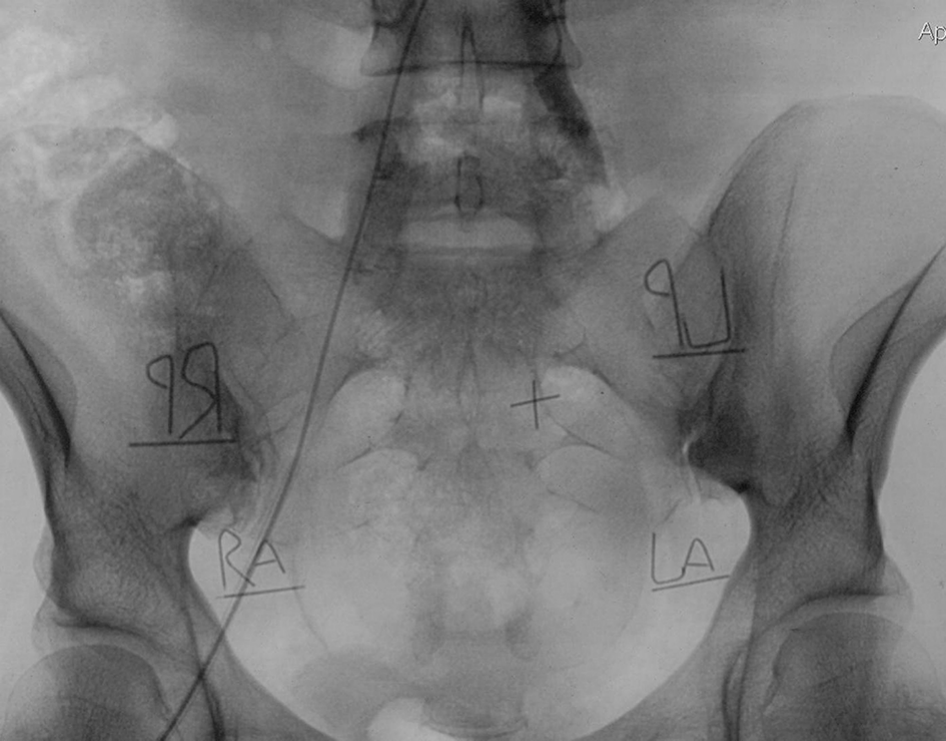
Markers showing TLD location in the pelvic cavity

As mentioned earlier, exposed dose to the ovaries was the main outcome measure of the study. Four dosimeter batches (each containing 3 TLD-100 chips) were used for each patient, two batches on the posterior surface ESD and two batches on the anterior surface of the pelvic region at the level of the right and left ovary. TLD batches were sealed by a waterproof and radiolucent cover and marked by a piece of wire and RA, LA, RP, LP markers (refers to right, left, anterior and posterior) to make their location detectable during the procedure (Figure 1). 

Configuration of the systems such as tube-table and patient to image receptor distance was set similar to what was used in the in-vitro study, although we did not interfere with the routine procedure and other parameters that were set by the internationist such as using magnifying and oblique views. The parameters of the procedure including KV and mA of fluoroscopy, frame rate, fluoroscopy time, KV and mAs and the number of spot images were filled during the procedure. After the procedure, TLD batches were removed and read by TLD-reader and the mean values of three TLD readings in each batch were used for calculating the radiation dose at their location. The depths of the ovaries from the posterior surface of the body were used individually for calculating the ovarian dose in each patient’s ovaries by using functions achieved in the in-vitro study (dose at the depth of interest). Since during some procedures, especially during magnifying and oblique views, the posterior TLD batches moved outside the radiation field making their results inaccurate, we had to use the result of anterior TLD-batches exit dose in the functions to calculate the depth dose.

### 3.5. Statistical Analysis

We used SPSS ver. 11.5 (SPSS Inc., Chicago, Il, USA) for statistical analysis. Comparison of the variables between the two groups was done by t-test or U-Mann Whitney after normality assessment of the data in the two groups. In addition, we compared the data of the two sides (right and left) considering the angiography system. All P values less than 0.05 were considered statistically significant.

## 4. Results

From January 2008 till May 2009, 30 patients were enrolled in this study of which 15 patients were assigned to the conventional angiography machine and 15 in the flat panel device. We had no patient loss after randomization.

The mean age, the mean weight and the mean height of the patients embolized in flat panel and conventional angiography groups were 37.7±6.3 years and 35.5±7 years (P=0.39); 66.3±12.4 kg and 64.9±10 kg (P=0.72); 161±8.3 cm and 160±6.6 cm (p=0.71), respectively.All procedures were technically successful in both groups and there were no complications.

The mean pelvic diameter was 21.4±3.2 cm in the conventional and 25.7±4.8 cm in the flat panel group (P=0.7).

The mean fluoroscopy time was 1212.8±646.7 seconds in the conventional and 1341±622 seconds in the flat panel group (P=0.58). The mean mAs of fluoroscopy was 10208±5976 in the conventional and 9277 ±6786 in the flat panel group (P=0.70).The mean spot number in the conventional group was 85.3± 34.8 and 177±91 in the flat panel group (P=0.001). The mean mAs of the spots was 2713±2050 in the conventional and 3173± 2862 in the flat panel group (P=0.62).The mean total mAs was 12921±6667 in the conventional and 11405±7160 in the flat panel group (P=0.36).

**Figure 2.**
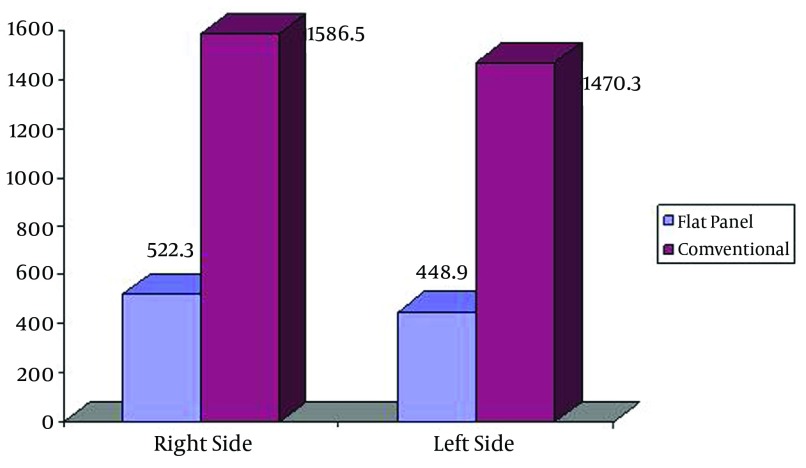
Comparison of entrance skin doses between the two systems on the right and left sides

The mean depth of the right ovary for patients embolized in flat panel angiography and conventional angiography was 12.7±2.5 cm and 11.3±2 cm, respectively (P=0.1). The mean depth of the left ovary for patients embolized in flat panel angiography and conventional angiography was 12.3±2.3 cm and 12±2.3 cm, respectively (P=0.76). The mean of the right side entrance dose was 1586.5±1221.4 mGy in the conventional and 522.3±400.1 mGy in the flat panel group (P=0.005). These figures were 1470±1170 mGy and 448.9±382.8 mGy for the left side, respectively (P=0.006). The mean of the right side exit dose was 18.8±12.3 mGy in the conventional and 9.4±6.4 mGy in the flat panel group (P=0.013) compared to the mean of the left side exit dose that was 16.7±11.3 mGy in the conventional and 10.2±7.2 mGy in the flat panel group (P=0.06).The mean of the right side ovarian dose was 139.9±92 mGy in the conventional and 23.6±16.2 mGy in the flat panel group (P<0.0001) compared to the mean of the left ovarian dose that was 101.7±77.6 mGy in the conventional and 24.6±16.9 mGy in the flat panel group (P=0.002) (Figure 2).

## 5. Discussion

One important point in interventional procedures such as UAE is to estimate and also to reduce the exposure dose. The gonads, which are in the direct path of the beam during embolization are one of the most sensitive organs to radiation. The possibility of infertility subsequent to radiation exposure during UAE needs further investigation.

Transient amenorrhea after this procedure is rare and persistent amenorrhea occurs in less than 1% (2, 3). Advanced maternal age adversely affects ovarian function; causing a decrease in the number of good quality oocytes and chromosomally abnormal conceptions that rarely develops further.

In one study, the authors assessedovarian function by measuring the serial basal follicle stimulating hormone (FSH) assay in 63 patients who had undergone UAE and concluded that most of their patients had no change in ovarian function after the procedure and in women over 45 years old, there was a chance of about 15% increase in basal FSH into the perimenopausal range (12).

There are many studies reporting normal ranges of pregnancy complications in the general obstetric population (13, 14). One study reported that the patient radiation exposure after UAE is up to double the dose level of diagnostic abdominal imaging such as multi-slice CT scan (15). The estimated ovarian dose after UAE is about 30-100 times higher than the dose of computed tomography of the trunk (0.1-1.9 cGy) and 12-30 times lower than the radiation dose after radiotherapy for Hodgkin disease of the pelvis (263-3, 500 cGy) (16). The extent of exposure relates to the tube voltage in KV, current time product (mAs), skin distance and the imaging techniques (17).

Increasing the experience of the interventional radiologist can lead to the decrease of the radiation dose (18, 19). Other factors that could contribute in dose reduction are radiographic techniques such as collimation, number and selection of projections and DSA image acquisition mode (19). Nikolic et al. (18) concluded that dose-reduction techniques, such as pulsed fluoroscopy, may reduce the ovarian dose (18). In one study, Broadhead et al. (20) compared digital and non-digital systems in performing barium studies and concluded that the dose is higher in non-digital systems compared to the digital systems.

Introducing the flat panel angiography systems not only causes increase in image quality, but also decreases the radiation dose to patients due to improvement in detective quantum efficiency (DQE) during interventional cardiology procedures (7, 9).

Some factors that might be contributed to dose reduction in the flat panel system are high DQE of the digital flat panel detector, use of real-time image information for optimization of digital image parameters by the system software and enhancement of dose efficiency by combination of a high-opacity tube and filtration.

Studies investigating the use of flat panel detectors in comparison with conventional angiography during interventional cardiology and transcatheter arterial embolization of hepatocellular carcinoma concluded that using the flat panel system can effectively reduce the radiation dose (8, 9, 21).

We did not find any study in the literature that compares these two systems in UAE, thus it is not possible to compare our results with those of other studies. In our study, the mean entrance dose in the conventional system group was significantly higher than that of the flat panel group and it was similar for the exit dose too. Indeed, we found that the mean ovarian dose in the conventional group was about five times higher than the flat panel group. This is especially important when we realize that most of the patients undergoing UAE are in fertile age and reducing the ovarian radiation dose is essential.

In conclusion, our results showed that using flat panel angiography system may be effective for reducing the ovarian radiation dose during UAE.
